# Influence of diabetes on cardiac resynchronization therapy in heart failure patients: a meta-analysis

**DOI:** 10.1186/s12872-015-0018-0

**Published:** 2015-03-21

**Authors:** Hui Sun, Yuqing Guan, Lei Wang, Yong Zhao, Hong Lv, Xiuping Bi, Huating Wang, Xuejing Zhang, Li Liu, Min Wei, Hui Song, Guohai Su

**Affiliations:** Department of Cardiology, Jinan Central Hospital, Affiliated with Shandong University, Jinan, Shandong Province 250013 China; Department of Geriatric Cardiology, Provincial Hospital Affiliated with Shandong University, Jinan, Shandong Province 250021 China

**Keywords:** Cardiac resynchronization therapy, Diabetes mellitus, Heart failure, Outcome, All-cause mortality

## Abstract

**Background:**

Diabetes mellitus is an independent risk factor of increased morbidity and mortality in patients with heart failure. Cardiac resynchronization therapy (CRT), a pacemaker-based therapy for dyssynchronous heart failure, improves cardiac performance and quality of life, but its effect on mortality in patients with diabetes is uncertain.

**Methods:**

We performed a meta-analysis of results from randomized controlled trials (RCTs) of the long-term outcome of cardiac resynchronization therapy for heart failure in diabetic and non-diabetic patients. Literature search of MEDLINE via Pubmed for reports of randomized controlled trials of Cardiac resynchronization for chronic symptomatic left-ventricular dysfunction in patients with and without diabetes mellitus, with death as the outcome. Relevant data were analyzed by use of a random-effects model. Reports published from 1994 to 2011 that described RCTs of CRT for treating chronic symptomatic left ventricular dysfunction in patients with and without diabetes, with all-cause mortality as an outcome.

**Results:**

A total of 5 randomized controlled trials met the inclusion criteria, for 2,923 patients. The quality of studies was good to moderate. Cardiac resynchronization significantly reduced the mortality for heart failure patients with or without diabetes mellitus. Mortality was 24.3% for diabetic patients with heart failure and 20.4 % for non-diabetics (odds ratio 1.28, 95% confidence interval 1.06–1.55; *P* = 0.010).

**Conclusions:**

Cardiac resynchronization therapy (CRT) may reduce mortality from progressive heart failure in patients with or without diabetes mellitus, but mortality may be higher for patients with than without diabetes after CRT for heart failure.

## Background

The strong association of diabetes mellitus (DM) and Heart failure (HF) has been well recognized for several decades. These two chronic medical conditions often coexist. The prevalence of DM in patients with HF approaches 30% [1]. DM contributes to the development and progression of HF and worsens the prognosis. Patients with both DM and HF have a 1.5- to 2-fold higher risk of death than non-diabetic patients with HF [2]. Despite the development of effective pharmacological and non- pharmacological treatments in recent years, the quality of life of many patients with HF is considerably impaired because of the frequent worsening of symptoms and continual poor prognosis, with sudden cardiac death as the major cause of death [3].

Cardiac resynchronization therapy (CRT) is an exciting therapy that can treat patients with systolic heart failure (HF) and left ventricular dysfunction who have a wide QRS complex. Fortunately, recent studies [4,5] have demonstrated the beneficial effect of cardiac resynchronization therapy (CRT) to improve symptoms, exercise capacity, and left ventricular (LV) systolic performance in HF patients with low ejection fraction and wide QRS complex. But the pathophysiology underlying HF in diabetic patients differs from that in nondiabetes. Reports on CRT in diabetic patients are limited and controversial. Whether CRT is equally effective for HF patients with or without DM is not well established.

We aimed to systemically review the literature to determine the effect of CRT on death in end-stage HF patients with and without DM.

## Methods

### Study search

We performed a literature search of MEDLINE via Pubmed for articles published from 1994 to 2012 describing RCTs of CRT for HF in patients with or without DM. RCTs had to include death as an outcome. Studies of CRT were first reported in 1994 [6]. The search was performed in January 2013. To focus on the chronic effects of CRT, we excluded reports of trials with <3-month follow-up. For multiple reports from the same trial, we used the most complete and/or recently reported data.

### Data abstraction

Two reviewers assessed report eligibility and abstracted data independently in an unblended [7] standardized manner. Abstracted data included eligibility criteria, baseline characteristics of patients, interventions, outcomes, and reported methodological quality (internal validity). The outcome of interest was death from any cause. Disagreements between reviewers were resolved by consensus.

### Data analysis

Because of relatively low event rates, odds ratios (ORs) closely approximated risk, and we collected OR data from reports. OR were pooled by use of a random-effects model with weighting based on inverse variance calculated according to DerSimonian and Laird [8]. Chi-square test was used to check for quantitative heterogeneity. We performed sensitivity analyses to determine the effect of changes in assumptions on the association of CRT and mortality. Publication bias was assessed with funnel plots. The analysis involved use of RevMan v4.2 (The Nordic Cochrane Center; Regshospitalet).

## Results

### Search results

**Figure 1:**
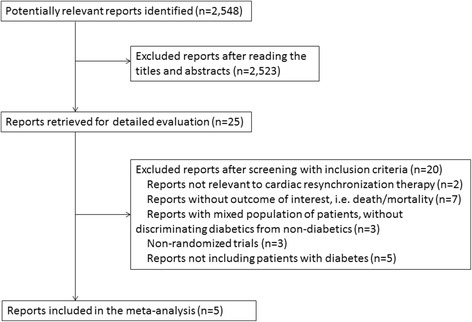
**Selective flowchart of studies included in the meta-analysis.**

The selection of reports is described in Figure 1. We identified 2,548 potentially relevant reports; 2,523 were excluded after reading the title and abstract. We retrieved the full-text versions of the remaining 25 reports, and only 5 were eligible for inclusion [9-13].

### Characteristics of patients

**Figure 2:**
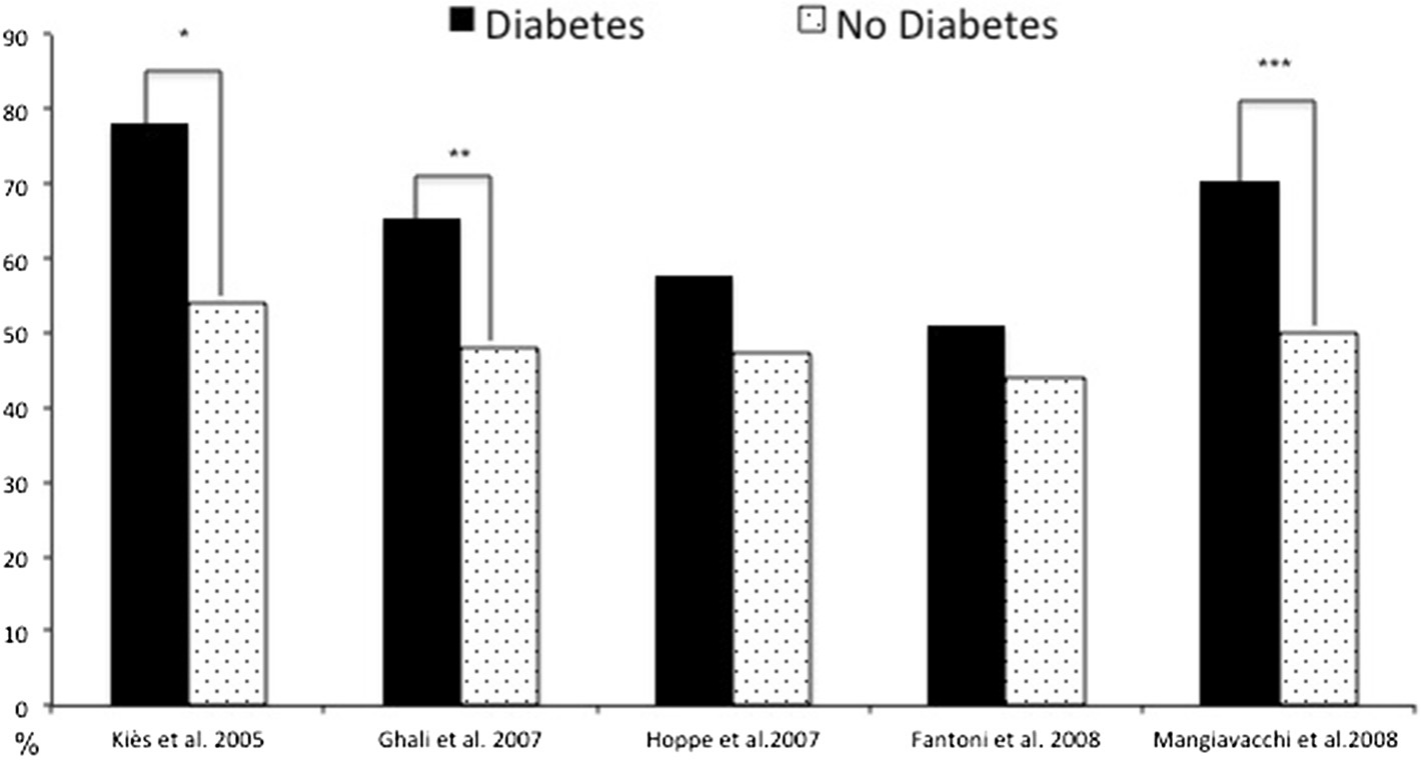
**Prevalence of coronary artery disease in patients with or without diatetes.** *P < 0.05, *P* < 0.01, *P* < 0.001.

Baseline patient characteristics (Table 1) were similar in the 5 trials [9-13]: at the time of CRT implantation, all patients had moderate to severe HF, with New York Heart Association (NYHA) functional class III or IV HF despite stable (>3 months) optimized medical therapy, severe left ventricular systolic dysfunction of any cause (ejection fraction ≤35%, end-diastolic diameter >55 mm) and ventricular conduction disturbances (QRS duration ≥120 ms). Mean ages ranged from 63 to 67 years and mean left ventricular ejection fraction from 21% to 28%. Most patients were men. For more than 54% of patients (2 trials), HF resulted from ischemic cardiomyopathy [9,10,13]. In contrast, in each of the 3 other trials, most patients had non-ischemic cardiomyopathies [11,12] (Figure 2). Baseline QRS duration, a measure of ventricular electrical dyssynchrony, was reported similarly prolonged for each of the 5 trials, with mean values ranging from 158 to 176 ms. Dyssynchrony was associated with left bundle-branch block for most patients in each trial. Baseline use of angiotensin-converting enzyme inhibitor or angiotensin receptor blocker was similarly high for all 5 trials and ranged from 88% to 98% (Table 1).

**Table 1:** **Characteristics of trials included in the meta-analysis**

	**Kiès et al. 2005** [9]	**Ghali et al. 2007 [2010]**	**Hoppe et al. 2007** [11]	**Fantoni et al. 2008** [12]	**Mangiavacchi et al. 2008 **[13]
No. of patients randomized	97	1211	813	355	447
Age, mean, y	63	66	67	63 ± 9	65.7 ± 9.7
Men, %	77	67	74	75	81
LVEF, mean, %	22	22	25	21.2 ± 6.2	28.9 ± 6.1
NYHA functional class, range	III-IV	III-IV	III-IV	III-IV	II-IV
Ischemic cardiomyopathy, %	62	55	40	47	54
QRS duration, mean, ms	176	158	160	163	166
ACEI inhibitor or ARB, %	88	88	95	98	99.3
insulin-treated diabetic patients, (%)	9 (28.1)	-	85 (10.5)	57 (40.4)	29 (31.9)
Follow-up, randomized, mo	6	12	36.4	35	36

**Figure 3:**
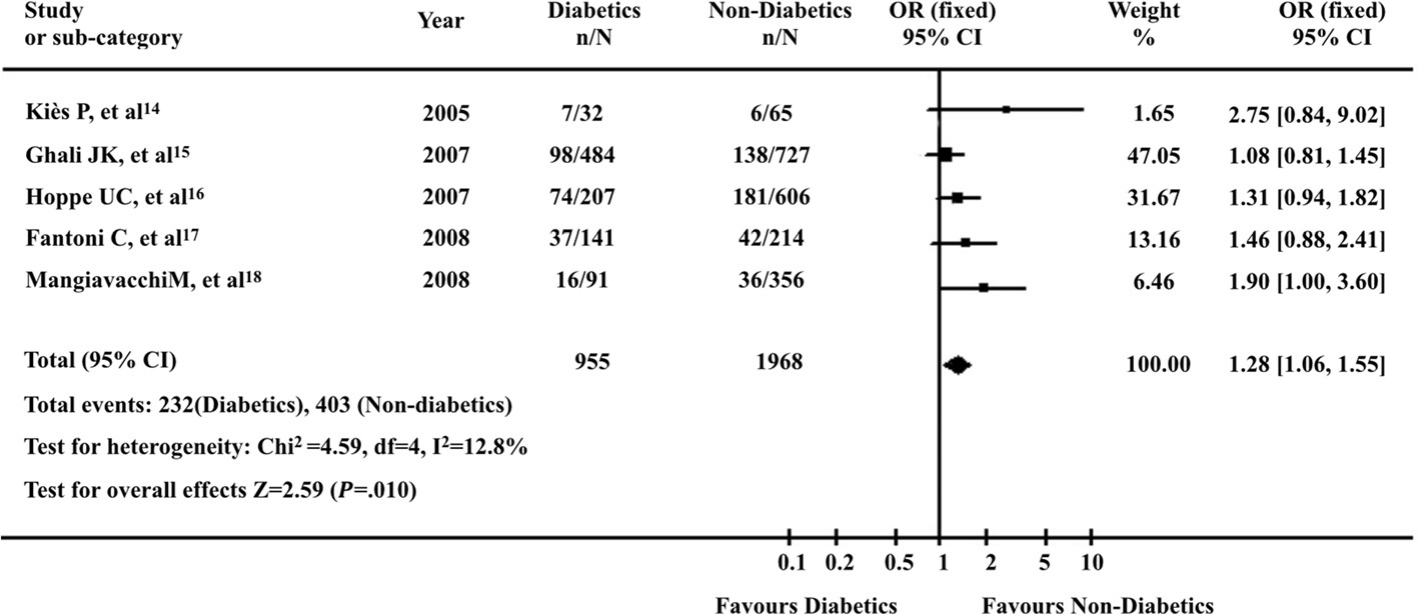
**Death among patients with cardiac resynchronization for heart failure for patients with or without diabetes.**

There is statistically significant increase in LVEF and 6-min walking test both in nondiabetic and in diabetic patients, with a trend toward a greater increase in the nondiabetic group cardiomyopathy after CRT [9,11-13]. CRT also reduced all-cause mortality in all patients with HF, regardless of presence of diabetes. For individual trials, mortality did not differ between diabetics and non-diabetics (Figure 3). However, on pooling data by a random-effects model, diabetes with HF was associated with a significant increase in death (OR 1.28; 95% confidence interval [95% CI] 1.06–1.55; *P* = 0.010). Pooled absolute rates of mortality during follow-up were 24.3% for HF patients with diabetes and 20.4% for nondiabetics. Chi-square test of ORs of mortality revealed no heterogeneity (*P* =0.33).

### Sensitivity analyses

We performed sensitivity analyses to determine the effect of changes in assumptions on the association of CRT and reduced mortality with HF and diabetes (Table 2). First, we compared fixed-effects and random-effects statistical models. The 2 types of models had similar results.

**Table 2:** **Sensitivity analysis of the effect of cardiac resynchronization on death in patients with and without diabetes**

	**No. of trials**	**No. of patients analyzed**	**Death from heart failure**
			**odds ratio (95% CI)**
Statistical model			
Random effects	5	2923	
Fixed effects	5	2923	1.57 (1.14-2.15)
NYHA baseline functional class			
II-IV	5	2923	1.28 (1.06-1.55)
III-IV	4	2476	1.24 (1.02-1.51)
Analysis with all studies except			
Kiès 2005 [9]	4	2826	1.26 (1.04-1.52)
Hoppe 2007 [11]	4	2514	1.27 (1.01-1.60)
Ghali 2007 [10]	4	1712	1.46 (1.14-1.87)
Fantoni 2008 [12]	4	2568	1.26 (1.03-1.54)
Mangiavacchi 2008 [13]	4	2476	1.24 (1.02-1.51)

Abbreviations: NYHA, New York Heart Association. Odds ratio, pooled odds ratio of death from all cause among patients with diabete or non-diabetes. Odds ratio more than 1.0 favors non-diabetes. 95% CI, 95% confidence interval.

Second, we analyzed data for only patients with NYHA functional class III and IV (moderate to severe HF) because the US Food and Drug Administration has approved CRT devices for such patients. The point estimate of the OR for death from HF for 2,476 class III and IV patients was similar to that for all 2,923 class II, III, and IV patients (1.24 vs. 1.28) (Table 2).

Third, we assessed the influence of individual trials on the pooled OR for death from HF. By excluding individual trials, the point estimates for the OR changed little and ranged from 1.24 to 1.46 (Table 2). Therefore, no single study had a major impact on the point estimate of the pooled OR.

### Publication bias

**Figure 4:**
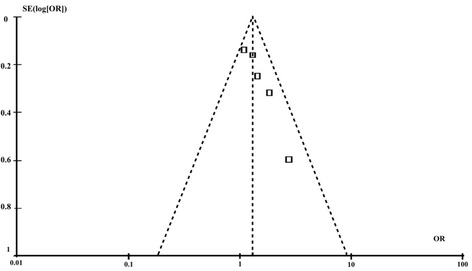
**Funnel plot of all studies in the meta-analysis of risk of all-cause mortality for patients with and without diabetes.** SE = standard error; OR = odds ratio.

The funnel plot for all-cause mortality during follow-up appeared symmetric, which suggested the absence of publication bias (Figure 4).

## Discussion

This meta-analysis of reports of CRT for HF in patients with and without diabetes was of a large cohort of patients undergoing CRT for chronic HF. We found a significant reduction in all-cause mortality for nondiabetic than diabetic patients. Thus, diabetic patients with advanced HF undergoing CRT may have an increased risk of mortality than nondiabetic patients. This increased risk of death seems to be due mainly to insulin-treated diabetes; the mortality estimates for nondiabetic patients and noninsulin-treated diabetic patients were similar, with the mortality estimate for insulin-treated patients substantially worse [13].

An important number of patients with HF have concomitant DM, for worse prognosis, likely because of the different underlying etiology of HF in patients with DM, who show increased hypertension, dyslipidemia and aggressive atherosclerosis. Previous reports suggested that diabetic patients were sicker than nondiabetic patients and presented a significantly higher prevalence of permanent atrial fibrillation and renal function impairment, larger left ventricles, and higher pulmonary artery systolic pressure [12]. These findings may be related to interstitial fibrotic tissue and alterations in myofibrillar proteins, which are frequently observed in diabetic patients. Accumulating deposition of interstitial fibrotic tissue and changes in myofibrillar proteins related to diabetes [14] might theoretically diminish the magnitude of reverse remodeling and the benefits on outcomes provided by CRT. These cardiac abnormalities, together with other major systemic changes induced by DM, may theoretically affect the efficacy of CRT.

The impact of diabetes on advanced HF in patients undergoing CRT was first addressed in a small study by Kies et al. [9]. After 6 months of CRT, response and long-term survival did not differ for patients with or without DM. Recently, the random controlled trial CARE-HF [11] showed that diabetic and non-diabetic patients undergoing CRT had a similar incidence of the combined end-point of death and unplanned hospitalisations for cardiac reasons. Regardless of the diabetes therapy and the presence of coronary artery disease, diabetes did not influence the beneficial effect of CRT on any endpoint. However, other research found diabetic patients with worse recovery of left ventricular ejection fraction over time (*P* = 0.057) and distance in the 6-min walking test (*P* = 0.018) than non-diabetic patients. Insulin-treated diabetes is associated with poor functional recovery and increased mortality in patients with advanced HF after CRT. Moreover, we found higher total mortality for patients with than without diabetes who had advanced HF and were undergoing CRT, independent of baseline characteristics. The increased risk of death for diabetic patients seems to be explained mainly by patients with insulin-treated diabetes [13].

Study suggested that patients with HF and DM (in particular insulin-treated DM) who undergo CRT seem to have worse prognosis than non-diabetic patients [9,11]. This finding may be due to a high incidence of ischemic etiology of HF and a limited effect of CRT on reverse left ventricle (LV) volumetric remodeling in these patients. The ischemic etiology of HF is an independent predictor of poor echocardiographic response to CRT. Patients with DM and HF have a relatively poor echocardiographic response to CRT [15], which may limit the effect of CRT on reverse LV volumetric remodeling in these patients. However, in 2 previously published CRT series, left ventricular dimensional and functional changes were similar for patients with and without DM [9]. This finding seems controversial, because patients with DM have a high incidence of coronary artery disease as an underlying etiology of HF. In several studies, ischemic etiology was found a negative predictor of reverse LV volumetric remodeling [16-18], which may be due to the progressive character of coronary artery disease and the presence of scar tissue not responsive to pacing [19]. So, the effect of diabetes on myopathic mechanisms and progression of cardiac dysfunction might influence the response of HF patients to CRT.

Recent observations suggest that diabetes treated or not with insulin has a different impact on mortality in advanced HF even after CRT [11,20]. Insulin use rather than diabetes may be the marker of adverse prognosis in patients with systolic HF. Patients not receiving insulin may at little or no increased risk of death. Insulin may be a marker of more severe and/or longer duration of diabetes and, therefore, risk of micro- and macrovascular complications of diabetes. The CARE-HF data are consistent with the association of insulin use or requirement and worse outcome rather than diabetes diagnosis. Appropriate RCTs are required to determine whether insulin is a marker or mediator of worse outcome.

In non-diabetic people, CRT significantly improves NYHA functional class and peak oxygen consumption and promotes ventricular reverse remodeling [21,22]. Similar significant changes were observed in diabetic patients [12], which confirms the cardiac and systemic improvements of CRT in patients with more severe abnormalities. Indeed CRT-induced improvements in NYHA functional class, daily spontaneous physical activity (evaluated by automatic monitoring of Activity-log Index from last-generation devices), and left ventricular end-systo/diastolic diameters were comparable in diabetic and non-diabetic patients. The trend toward lower baseline peak oxygen consumption and larger ventricular dimensions in diabetic versus non-diabetic patients indirectly confirms the greater severity of illness in the diabetic group. Left ventricular ejection fraction and oxygen consumption at peak were significantly greater in non-diabetic than diabetic patients with CRT, which suggests better improvement of functional capacity after CRT in non-diabetic patients.

## Conclusions

This meta-analysis suggests that diabetic patients with advanced HF undergoing CRT exhibit higher total mortality than nondiabetic patients. The increased risk of death of diabetic patients with HF seems to be mainly explained by patients with insulin-treated diabetes. The survival estimates for nondiabetic and noninsulin-treated diabetic patients were similar, whereas that for insulin-treated patients was substantially worse. Intriguingly, cardiac death accounted for most of the deaths in patients without diabetes, but a relevant proportion of the excess mortality of patients with diabetes seemed to result from noncardiac causes. CRT had a beneficial effect on reverse remodeling in all patient groups (without diabetes, with noninsulin-treated diabetes, with insulin-treated diabetes), with a slight but not statistically significant increased improvement in ejection fraction in nondiabetic patients. However, diabetic patients had a smaller increase in exercise tolerance over time. This finding may reflect the presence of vascular and peripheral neurological disease and less-efficient muscular metabolism in this population or more severe diastolic impairment in diabetic patients, [12] Future research could investigate possible “peripheral” versus “central” factors that may explain why diabetic patients present less functional improvement during follow-up. Although insulin-treated diabetic patients shows poorer survival than the other 2 patient groups, we cannot conclude that insulin-treated diabetic patients do not benefit from CRT because of the absence of a control group without CRT in the studies. A recent analysis of data from the CARE-HF trial, including a control group, showed that insulin-treated diabetic patients had a markedly worse prognosis than nondiabetic patients and that CRT was equally effective in both patient groups in reducing mortality.

In our group of advanced HF patients, CRT significantly improved functional capacity, promoted reversal of the maladaptive remodeling process, and reduced the sympathetic drive to the heart in both diabetic and non-diabetic patients, over a long period of time. Consistently, morbidity and mortality were comparable between diabetic and non-diabetic CRT patients. Further evidence to support or refute the negative prognostic role of diabetes in CRT patients is now required.
